# Haemolymph removal by *Varroa* mite destabilizes the dynamical interaction between immune effectors and virus in bees, as predicted by Volterra's model

**DOI:** 10.1098/rspb.2019.0331

**Published:** 2019-04-17

**Authors:** Desiderato Annoscia, Sam P. Brown, Gennaro Di Prisco, Emanuele De Paoli, Simone Del Fabbro, Davide Frizzera, Virginia Zanni, David A. Galbraith, Emilio Caprio, Christina M. Grozinger, Francesco Pennacchio, Francesco Nazzi

**Affiliations:** 1 Dipartimento di Scienze AgroAlimentari, Ambientali e Animali, Università degli Studi di Udine, Udine, Italy; 2 School of Biological Sciences, Georgia Institute of Technology, Atlanta, GA, USA; 3 Dipartimento di Agraria ‘Filippo Silvestri’, Università degli Studi di Napoli ‘Federico II’, Portici (Napoli), Italy; 4 CREA, Council for Agricultural Research and Economics, Research Center for Agriculture and Environment, Bologna, Italy; 5 Department of Entomology, Center for Pollinator Research, Huck Institutes of the Life Sciences, Pennsylvania State University, University Park, PA, USA

**Keywords:** *Apis mellifera*, *Varroa destructor*, deformed wing virus, Volterra equations, host–parasite interactions

## Abstract

The association between the deformed wing virus and the parasitic mite *Varroa destructor* has been identified as a major cause of worldwide honeybee colony losses. The mite acts as a vector of the viral pathogen and can trigger its replication in infected bees. However, the mechanistic details underlying this tripartite interaction are still poorly defined, and, particularly, the causes of viral proliferation in mite-infested bees. Here, we develop and test a novel hypothesis that mite feeding destabilizes viral immune control through the removal of both virus and immune effectors, triggering uncontrolled viral replication. Our hypothesis is grounded on the predator–prey theory developed by Volterra, which predicts prey proliferation when both predators and preys are constantly removed from the system. Consistent with this hypothesis, we show that the experimental removal of increasing volumes of haemolymph from individual bees results in increasing viral densities. By contrast, we do not find consistent support for alternative proposed mechanisms of viral expansion via mite immune suppression or within-host viral evolution. Our results suggest that haemolymph removal plays an important role in the enhanced pathogen virulence observed in the presence of feeding *Varroa* mites. Overall, these results provide a new model for the mechanisms driving pathogen–parasite interactions in bees, which ultimately underpin honeybee health decline and colony losses.

## 1. Introduction

Efficient pollination is vital for crop production [1] and the honeybee is the prevailing managed insect crop pollinator. Honeybees suffer from a range of adverse factors [2]; in particular, the deformed wing virus (DWV) is implicated in the substantial colony losses reported in many parts of the world [3] and the parasitic mite *Varroa destructor* plays a key role in virus transmission and replication [4,5]. Moreover, previous studies have demonstrated that the spread of *V. destructor* contributed to turning a widespread viral infection into a devastating epidemic [3].

The capacity of the *Varroa* mite to transfer DWV was proved by Ball [6] and later confirmed under field conditions [7]; these authors also provided preliminary evidence for the replication of the virus within the mite, which was later confirmed [8]. However, the mite does not act only as a vector of the virus, thus increasing the pathogen's prevalence, but can also trigger uncontrolled replication in infected bees, which undermines colony survival [9]. Several mechanisms have been proposed to explain the role of the mite as an activator of the virus, based on experiments or samplings carried out under different settings and variable conditions. Initially, increased replication was attributed to a direct immune-suppressive action exerted by the mite [10]. Based upon field experiments aiming at assessing the impact of *Varroa* infestation on bees, we showed that the immune challenge represented by the feeding mite amplifies existing viral infections through an escalating bee immunosuppression, perpetuated by the increasing DWV abundance [9]. Two additional mechanisms accounting for the higher viral load observed in mite-infested bees were proposed. First, it was suggested that higher infection levels, leading to crippled winged bees, are linked to the active replication of the virus within the infesting mite [8]. Second, based on the study of a region of the RNA-dependent RNA polymerase (RdRp) gene of DWV, during a *Varroa* invasion into a previously mite-free area, the possibility that the mite can select for a single virulent strain adapted to mite transmission, was proposed [11]. This facilitation seems to take place also at the individual level when a mite infests a honeybee, where either parasitization or artificial injection favours the replication of a single quasi-clonal DWV strain within the bee [12].

However, the available data can also support additional models on how mite feeding can influence the viral titre in bees. In particular, the significant increase in the viral titres in bees infested by three mites versus a single mite [9] and previous observations about the effects of multiple mite infestations on the proportion of symptomatic bees [13] suggests that feeding intensity may play a role. This could be the result of the injection of increasing amounts of mite derived immune suppressing factors into the bee's haemolymph [14]. However, when more *Varroa* mites parasitize the same bee, they make a single wound into the bees' cuticle to access the haemolymph and feed from the same opening [15,16], thus likely eliciting the same response in terms of melanisation and clotting, but subtracting a substantially higher volume of haemolymph. This, in turn, could be responsible for the increased viral replication observed in case of multiple infestation. The possible role of haemolymph removal on DWV dynamics seems to be confirmed by the proliferation of DWV that can be observed after simple wounding with capillary needles and the resulting bleeding from the open wounds [17]. The mite feeding on honeybee fat body rather than on haemolymph, recently claimed [18], does not challenge the established view that this parasite feeds upon the internal fluids, which could well be enriched with nutrients released by extra-oral digestion of fat body.

On a purely theoretical background, it is possible to hypothesize that the concurrent removal of virus particles and circulating antiviral immune effectors by the blood-feeding mite can generate a dynamic response similar in principle to that observed when both prey and predators are constantly removed from a predator–prey system [19]. This apparently counterintuitive circumstance was first explained by Volterra, at the beginning of the last century [19], for describing the unexpected fluctuations of certain fish species in the Adriatic Sea. The proposed model clearly showed that the subtraction of both predators and prey, through fishing, could result in the proliferation of the prey [19].

In summary, in spite of the large body of evidence about the effect of mite infestation on the dynamics of viral infection in the honeybee and the importance of the *Varroa*–DWV association for honeybee health, there are still multiple hypotheses on the major mechanisms underpinning the higher viral load observed in mite-infested bees, that are not mutually exclusive. In this study, to further contribute to the analysis of the mechanisms underlying the viral proliferation in mite-infested bees, we carried out controlled laboratory experiments to test the hypothesis that mite feeding ‘per se’ can destabilize viral immune control through the removal of both viral ‘prey’ and immune ‘predators’, triggering viral replication. We assess the impact of controlled bleeding on viral proliferation; we also evaluate if the resulting viral load is in part or totally owing to any of the other mechanisms described in the literature. This type of micro-ecological analysis of host–pathogen interactions has broad implications in the research area of animal parasitology.

## 2. Results

### (a) Viral infection in mite-infested honeybees

**Figure 1.**
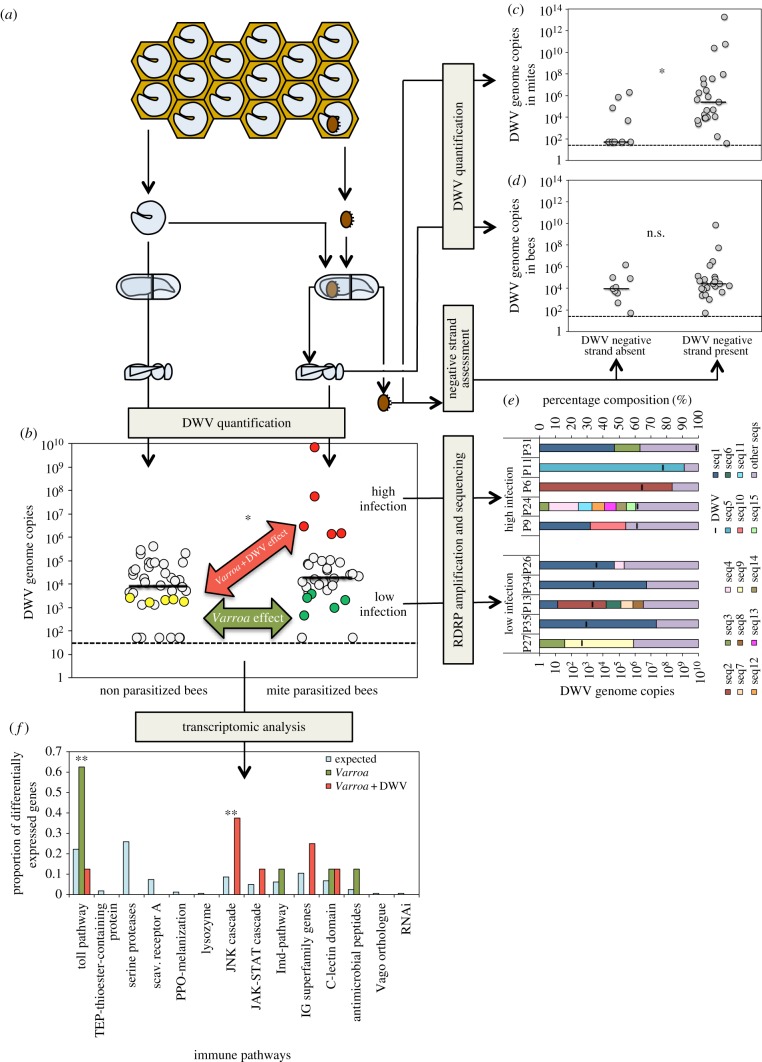
Evaluation of existing hypotheses about the role of *Varroa* mite in increasing virulence of DWV: methods and results. (*a*) Individual bees naturally infected with DWV were artificially infested with one *Varroa* mite or left uninfested. (*b*) Viral load in individual bees infested with one mite or left uninfested as a control. In this and following similar figures, the dashed line represents the lower detection limit for the methodology used; the solid lines represent the median viral load. The samples used for the transcriptomic analysis are marked with different colours: yellow (uninfested-low virus infected bees), green (mite-infested-low virus infected bees) and red (mite-infested-high virus infected bees). An asterisk marks a significant difference at *p* < 0.05. (*c*) DWV genome copies in *Varroa* mites where an active replication was detected (DWV negative strand present) or not (DWV negative strand absent). An asterisk marks a significant difference at *p* < 0.05. (*d*) DWV genome copies in bees infested by mites where an active replication was detected (DWV negative strand present) or not (DWV negative strand absent). (*e*) Prevalence of different DWV variants in infected bees with variable virus infection levels. The thick vertical lines represent DWV genome copies observed in each sample. (*f*) Effect of the *Varroa* mite and the combination *Varroa*-DWV on the expression of genes of the canonical immune pathways. The proportion of differentially expressed genes in each pathway, as resulting from the comparison: uninfested-low viral infected bees versus mite-infested-low viral infected bees (i.e. *Varroa* effect) and from the comparison: uninfested-low viral infected bees versus mite-infested-high viral infected bees (i.e. *Varroa* + DWV effect), is reported as well as the proportion of immune genes belonging to that pathway (i.e. expected). Two asterisks mark significant differences at *p* < 0.01 between expected and observed proportions.

To clarify the role of the mite in the dynamics of viral infection in honeybees, we evaluated the presence and abundance of DWV in adult bees that were artificially infested with one mite as mature larvae or were not infested with mites as controls (figure 1*a,b*); viral presence and titres were evaluated using quantitative real-time polymerase chain reaction (qRT-PCR) with sequence-specific DWV primers. Furthermore, a subset of these bees (figure 1*b*) was subjected to next generation sequencing (NGS) which allowed us to confirm that the bees were infected with DWV and the sequences were greater than 98% identical with a published sequence obtained from a sample collected in the same apiary in 2006 (i.e. NC_004830.2; electronic supplementary material, figure S1) and clearly separated from other genotypes of DWV (i.e. NC_006494.1) or recombinants that were associated with higher virulence in other studies (electronic supplementary material, figure S1) [12,20]. In particular, sequencing revealed that the viral genotype present in this area can be regarded as DWW type A [21,22].

We found that 80% of individuals not exposed to mite feeding (*n* = 40) were DWV infected. However, the prevalence of DWV in bees infested by a DWV-infected mite (*n* = 27) was higher at 96% (electronic supplementary material, figure S2; *χ*^2^_1_ = 3.681, *p* = 0.055).

Viral load was higher in bees parasitized by mites compared to control bees (figure 1*b*; median viral load in mite-infested bees (*n* = 32) = 1.91 × 10^4^ DWV genome copies; median viral load in uninfested bees (*n* = 40) = 8.06 × 10^3^ DWV genome copies; Mann–Whitney *U* = 482, *n*_1_ = 40, *n*_2_ = 32, *p* = 0.037). DWV infection levels in uninfested bees showed a great variability ranging from 10^3^ to 10^6^ DWV genome copies per bee (figure 1*b*). However, DWV infection levels showed even greater variability in mite-infested bees; in fact, most mite-infested bees showed infection levels falling within the same interval as that recorded in uninfested bees, but a few specimens largely exceeded the upper limit of this interval, reaching 10^10^ viral genome copies per bee (figure 1*b*). Consequently, the distribution of viral loads was very skewed in mite-infested bees (skewness of the distribution of viral loads in mite-infested bees (*n* = 30) = 5.48, skewness in uninfested bees (*n* = 32) = 2.50). Individual bees sampled later in the field season, when the DWV prevalence and the basal infection rate are higher [9], and artificially infested with one mite, showed a similar skewed distribution of infection levels, with some individuals displaying very high DWV infection levels (skewness of the distribution of viral loads in mite-infested bees (*n* = 58) = 5.66; electronic supplementary material, figure S3). Moreover, re-evaluation of previous data demonstrating the effect of single and multiple mite infestations on viral loads in bees [9] revealed a similar underlying distribution, with a higher median viral infection in mite-infested bees and the distribution of viral loads becoming increasingly sparse (electronic supplementary material, figure S4).

In summary, the DWV infection data show that the higher viral load observed, on average, in *Varroa*-infested bees is owing to a change in the distribution of individual viral levels, which is right skewed, owing to the presence of a sub-population of highly infected bees. Similar results were previously observed using a different experimental approach [12].

### (b) Viral replication in mites

To study the vector role of *Varroa*, we evaluated the mites infesting the experimental bees above (figure 1*a*) and found that their infection levels were generally higher than those in the bees themselves (median viral load in mites (*n* = 32) = 4.60 × 10^04^). A significant correlation was found between the mites' viral load and viral load of the bees they infested (electronic supplementary material, figure S5; *n* = 32, Spearman corr. coeff. = 0.531, *t*_30_ = 3.433, *p* = 0.002). However, this result cannot be unequivocally interpreted, because the observed correlation could be owing either to the fact that a highly infected mite, harbouring an intense viral replication, can inject higher amounts of viral particles, or that a mite infesting a highly infected bee can acquire more virus while feeding.

Active replication of single-stranded positive RNA viruses results in the synthesis of the complementary negative strand that is used as a template for the production of viral copies. Therefore, to assess the importance of viral replication within the mite on the level of bee infection, we assessed the presence of DWV negative strands in the mites used for the artificial infestation of bees (figure 1*a*). As expected, the mites containing DWV negative strands had a significantly higher infection level than those where no negative strands were found (figure 1*c*; Mann–Whitney *U* = 42, *n*_1_ = 9, *n*_2_ = 23, *p* = 0.005). However, when we examined whether the viral replication in the parasite was related to the viral load in the host, we found that the infection level of bees infested by mites where an active viral replication was detected was not significantly different from that measured in bees infested by mites which did not apparently harbour an actively replicating virus (figure 1*d*; Mann–Whitney *U* = 80, *n*_1_ = 9, *n*_2_ = 23, *p* = 0.157).

### (c) Composition of the viral mutant cloud

Short replication time and limited correction capability in RNA viruses favour rapid genetic changes, so that, even in a single host, a virus population normally consists of an ensemble of different genetic sequences. Previous studies focusing on the viral RdRp highlighted an important effect of mite parasitization on viral diversity [11]. Therefore, to assess the importance of this factor in the higher viral load observed in mite-infested bees, we amplified and sequenced by NGS the viral region encoding the virus RdRp, in five highly infected bees and five bees with low infection levels (average DWV genome copies per bee of 1.41 × 10^9^ and 1.95 × 10^3^, respectively) that were obtained from the previous experiment (figure 1*b*). From 74 to 559 different variants were reconstructed in each sample, based on a number of viral reads ranging from 40 107 to 160 842 (electronic supplementary material, data S1). We found no obvious common sequence in low versus high virus infected bees: the most represented sequence was present in six samples from both the low and highly infected groups, at prevalences ranging from 11 to 74% (figure 1*e*; electronic supplementary material, data S1). Thus, a link between viral load and molecular diversity was not found, at least at the level of RdRp sequence variation (figure 1*e*; electronic supplementary material, figure S6).

### (d) Effects of mite infestation and viral infection on the transcriptome of honeybees

To disentangle the effect of *Varroa* mite parasitization from that of DWV infection on the immune response of bees, we studied the expression of immune genes in bees exposed to a different combination of stress factors (figure 1*b*; electronic supplementary material, data S2). In particular, to assess the influence of the mite (i.e. *Varroa* effect), we compared the expression level of immune genes in five uninfested bees bearing a low viral infection (average DWV infection = 2.04 × 10^3^, yellow circles in figure 1*b*) and five mite-infested bees bearing a similar low viral infection level (average DWV infection = 1.95 × 10^3^, green circles in figure 1*b*). Next, to assess the influence of the combination *Varroa*–DWV (i.e. *Varroa* + DWV effect), we compared five uninfested bees bearing a low viral infection with five mite-infested bees bearing a high viral infection level (average DWV infection = 1.41 × 10^9^, red circles in figure 1*b*).

We found that different immune pathways were differentially affected by *Varroa* mite alone and the replicating virus in the presence of the mite (figure 1*f*; electronic supplementary material, data S2). Overall, infestation with mites, at low viral infection levels, caused significant changes in expression (i.e. upregulation) of genes involved in the Toll pathway, while very high DWV infection levels associated with *Varroa* infestation caused significant changes in expression of genes involved in the JNK pathway (figure 1*f*; electronic supplementary material, data S2), although this latter causal link is not as strong as the former since it may result both from an effect of high viral infection on immune expression and vice versa. Thus, the impact of *Varroa* mite feeding on bee immune response is different from the impact of the high viral titre stimulated by the mite. Furthermore, this experimental design, allowing the separation of the mite effect from that of the virus, confirmed that immune suppression by the mite [10] did not play a major role under these conditions.

### (e) Immune-virus ‘predator–prey’ dynamics within the host

In 2012, we proposed a series of mathematical models describing how within-host viral dynamics are controlled by the immunological response, which in turn can be modified by the presence of the virus and other stress conditions, such as mite feeding or pesticide exposure [9,23]. The simplest model consistent with the observation of divergent outcomes (low-cryptic or high-overt infection) required a threshold immune-suppressive effect of DWV. Given this assumption, any factor that depletes the immune system (e.g. increasing mite load) will lead to a gradual increase in a stable DWV set-point until, for sufficiently large depletion, a critical transition to unbound viral replication will follow, leading to overt symptoms and ultimately host death. We hypothesized that, in case of mite infestation, immune depletion may result from the activation of competing immune reactions cross-modulated by shared networks of transcriptional control and, in particular, the melanisation and clotting reactions triggered at the mite's feeding site, which are under the control of a NF-kB transcription factor that is involved also in antiviral response [9,24].

In the electronic supplementary material, figure S7, we replicate the theoretical analysis from [9], illustrating that the low stable viral equilibrium and the high unstable equilibrium (solid and dashed black lines, main figure) converge as the extent of immune depletion *y* increases, ultimately leading to unconstrained growth of viral titre.

In the current analysis, we now examine the impact of perturbations around the low stable equilibrium. Specifically, we ask: what happens to the coupled viral and immunological dynamics when initially stable levels of *V* and *I* are transiently perturbed away from their stable equilibrium by the loss of haemolymph? The two grey dots in the electronic supplementary material, figure S7 represent a 20% drop in haemolymph volume under different initial bee health settings (differing values of *y*). This quantity, consistent with one mite and its offspring feeding over 12 days, was evaluated from available data on mite feeding during the reproductive phase [25] and total haemolymph volume [26]. The two inset time-series diagrams illustrate that, while the healthier bee (lower *y*) returned to its prior equilibrium state (left inset diagram), the less healthy bee (higher *y*) was driven into the unstable runaway regime by the same proportionate degree of haemolymph loss. These results illustrate that the simultaneous removal of both virus and immune effectors can lead to the destabilization and subsequent runaway increase in viral titre.

### (f) Haemocytes as antiviral barriers in honeybee's haemolymph

In the model above, we assumed that loss of haemolymph results in a perturbation of the levels of both viruses and antiviral immune effectors contained in the bee's blood. To confirm this assumption, we first analysed the honeybee's haemolymph by qRT-PCR and found 10^3^–10^8^ DWV particles µl^−1^. Then, to demonstrate that haemocytes play an important role as antiviral barriers in the haemolymph, we engaged the circulating haemocytes in an intense cellular immune reaction and measured the impact of haemocyte depletion on viral replication, similarly to as recently performed in *Drosophila melanogaster* [27]. The injection of chromatographic beads into white-eye bee pupae, naturally infected by DWV, resulted in an intense encapsulation response by haemocytes (electronic supplementary material, figure S8*a*), which was associated with a concurrent increase in viral load (electronic supplementary material, figure S8*b*; Mann–Whitney *U* = 15, *n*_1_ = *n*_2_ = 10, *p* = 0.004). This suggests that in bees, like in flies, the depletion of functional haemocytes negatively affects the antiviral defence barriers and demonstrates the important role of these cells as antiviral effectors.

### (g) Effects of the increasing haemolymph subtraction on viral proliferation

**Figure 2.**
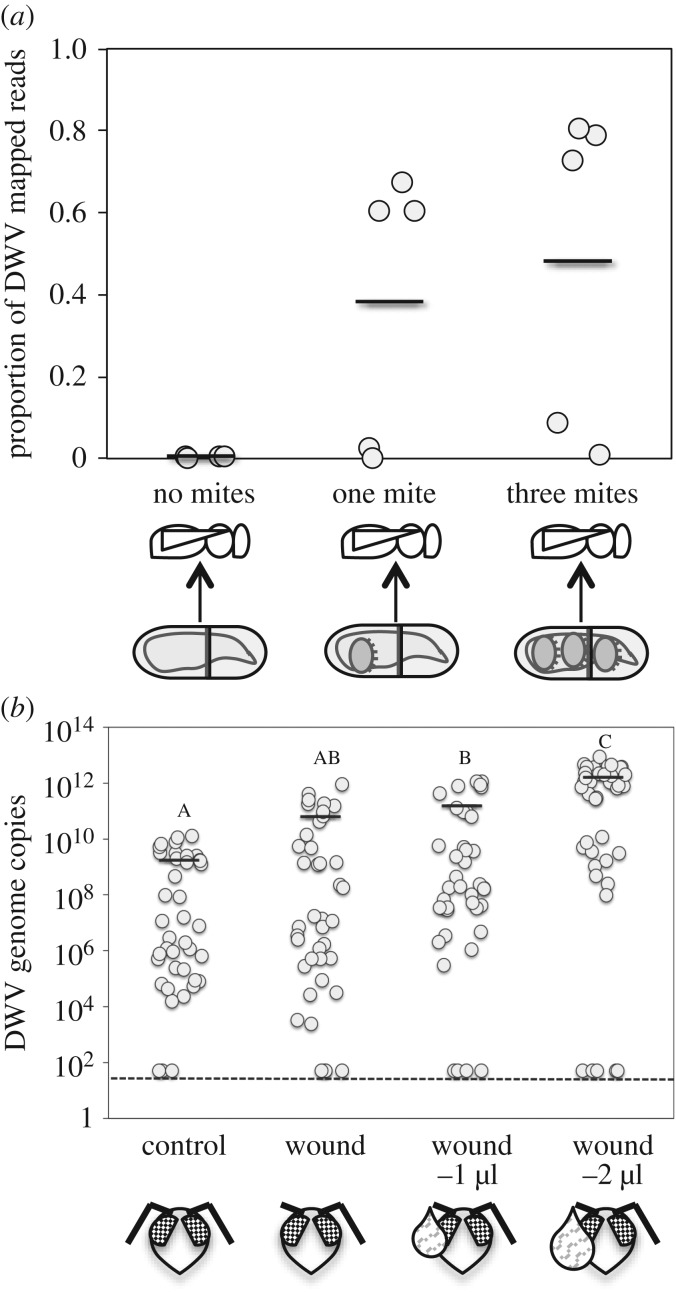
Increased feeding by *Varroa* mite as well as increased subtraction of haemolymph cause increased DWV infection in bees. (*a*) Viral load, as the proportion of reads mapping to DWV genome, in naturally infected bees artificially infested with no mites, one mite or three mites; the horizontal solid lines represent the average viral load. (*b*) The number of DWV genome copies in naturally infected bees after the removal of 1 or 2 µl of haemolymph through a wound is reported along the corresponding viral infection in control bees and wounded bees with no haemolymph subtraction. Different letters mark experimental groups significantly differing from each other (*p* < 0.001); consistently with the statistical analysis used here, the horizontal solid lines represent the average viral load.

To verify the hypothesis that haemolymph subtraction can trigger viral proliferation by perturbing the dynamics of virus and immune effectors, we carried out another laboratory experiment by artificially infesting mature bee larvae with one mite or three mites, using non-infested bees as controls, and by assessing both the viral infection level and immune response at eclosion by RNAseq. We observed that higher DWV titres are associated with heavier mite infestations (figure 2*a*; electronic supplementary material, data S3; Kruskal–Wallis: *H*_2_ = 6.41, *p* = 0.041) and likely with the removal of higher amounts of haemolymph, in accordance with the results reported above (electronic supplementary material, figure S4). The lack of a differential immune response in multiple versus single mite-infested bees observed in this case suggests that haemolymph loss, rather than an increasing mite-induced immune suppression, can generate an increasing level of viral infection (electronic supplementary material, figures S9A,B and data S3; note that no differentially expressed genes were found in the comparison: one versus three mites, whereas 66 and 50 differentially expressed genes were found, respectively, from the comparisons: no mite versus one mite and no mite versus three mites).

The possibility that the removal of increasing amounts of haemolymph could have affected the cross-talk between metabolism and immunity, as a consequence of nutrient subtraction, seems to be ruled out by the transcriptional data, since the genes involved in nutrient use and metabolism are not significantly differentially regulated across the treatment groups (electronic supplementary material, data S4). Furthermore, the analysis of the whole transcriptome of the bees used in this experiment does not support the occurrence of dramatic physiological changes associated with haemolymph removal [28].

To further corroborate our hypothesis, we assessed the impact of haemolymph subtraction in the absence of mite feeding by comparing viral replication in naturally infected bee pupae from which different amounts of haemolymph were removed with a microcapillary tube from a cut antenna, using wounded or untreated bees as controls. Our results demonstrated that viral load varied across treatments, with a clear dose-dependent response, positively linking the volume of removed haemolymph to the viral titre measured using quantitative real-time PCR 4 days after bleeding (figure 2*b*; χ^2^_3_ = 107.34, *p* < 0.001). In particular, the viral infection in bees to which 2 µl of haemolymph were removed was approximately 10 times higher than that observed in bees which had only 1 µl of haemolymph removed from a single wound (figure 2*b*; Tukey's test, *p* < 0.001), suggesting that subtraction alone can play a role.

This quantity of haemolymph is consistent with the amount that a mite can subtract in about 1 day [25,26] and probably insufficient to trigger a metabolic syndrome related to nutrient subtraction. At this time point (i.e. 4 days after bleeding), a significant decrease in the expression of *Dorsal 1A*, a gene encoding a protein in the NF-kB family, indicating an active immune suppression by the DWV [9], was observed in the bees belonging to the experimental groups which had haemolymph removed (electronic supplementary material, figure S10; Mann–Whitney *U* = 65, *n*_1_ = 20, *n*_2_ = 20, *p* < 0.001).

Four days later, the viral infection was similar to control in all experimental groups apart from the one which had 2 µl of haemolymph removed (electronic supplementary material, figure S11). This is consistent with the long-term immune suppression and related unbounded viral replication ensuing after a critical threshold of viral titre is surpassed, as predicted by our model [9].

## 3. Discussion

Several mechanisms have been proposed to explain the higher viral load observed in bees infested by the *Varroa* mite. However, the predictions of those models are not supported consistently across experiments, including the ones performed here. In this study, we propose a micro-ecological model based on the destabilization of virus population and immune effectors by the removal of haemolymph; this mechanism, which is strongly supported by our results, is not mutually exclusive to the previous models, but complements them well.

DWV prevalence in uninfested bees (i.e. 80%) is consistent with available data about DWV infection in honeybee eggs and larval food [29–31] and clearly indicates that trans-ovarial and trans-stadial transmission, as well as viral acquisition by feeding upon contaminated food during the pre-imaginal life, play an important role in the spread of DWV infection within the hive (electronic supplementary material, figure S12). The higher proportion of infected bees among those infested by a mite, together with the presence of replicating viruses within the mites, confirms the role of *V. destructor* as a vector of the virus (electronic supplementary material, figure S12). More importantly, our results highlight the fundamental role of the mite for the increased virulence of DWV in infected honeybees. Collectively, our experimental data allow us to conclude that, under the conditions of our experiment, the capacity of the mite to host the viral pathogen replication [8] (electronic supplementary material, figure S13A) appears to be of limited importance for the dynamics of DWV infection in bees. The similar composition and structure of the mutant clouds, observed in low and highly infected bees, do not support an important role of viral diversity at the level of RdRp in the modulation of observed levels of DWV virulence at individual level (electronic supplementary material, figure S13B), as proposed earlier [11,12] but recently questioned [32]. Our transcriptomic study further suggests that immunosuppression by the mite [10] (electronic supplementary material, figure S13C) does not play an important role, as previously proposed [17]. Instead, on the basis of our experimental and theoretical results, we conclude that the stress resulting from mite feeding has the potential of destabilizing the equilibrium between the pathogen and the bee's immune control [9] (electronic supplementary material, figure S13D). Here we confirm our previous hypothesis, based on the depletion of a shared immune resource [9,24] and further show that the intensity of mite feeding can affect the progression of viral infection through a dynamic process triggered by the concurrent removal of the virus and antiviral effectors, which is well described by models proposed for predator–prey interactions (electronic supplementary material, figure S13E).

In 1926, the mathematician Vito Volterra, to explain the unexpected fluctuations of certain fish species in the Adriatic Sea, developed his famous model, which clearly showed that the subtraction of both predators and prey, through fishing, could result in the proliferation of the latter [19]. Here we suggest that the pure subtraction of haemolymph—containing both virus and immune factors—from the host, by the feeding mite (electronic supplementary material, figure S13E), could trigger the proliferation of DWV which can be sustained by the depletion of a shared immune resource [9,24] and progressively reinforced by the viral-induced immunosuppression taking place as soon as the pathogen surpasses a critical threshold [9].

The model we propose here implies that haemolymph contains both virus and immune effectors whose density can be altered by the feeding activity of the mite. The presence of the virus within the circulating haemolymph is confirmed by our data and the significant correlation between viral infection in bees and the mites which fed upon them. Furthermore, several possible proteins and cells can act as antiviral effectors circulating in the haemolymph of bees and other insects. In particular, circulating antimicrobial peptides certainly play a still uncharacterized role in the immune response to viruses, being constantly upregulated upon virus infection [33]. In *Drosophila*, convincing evidence has been recently provided regarding the contribution of haemocytes to antiviral defence through phagocytosis [27] and the involvement in RNAi [34]. Our observation that higher viral loads can be observed after engaging the circulating haemocytes in an intense cellular immune reaction suggests that haemocytes play a similar role in the antiviral response of honeybees.

In general, our conceptual hypothesis represents the most parsimonious interpretation of the mite role in the enhanced virulence of the virus and provides the logical framework for future experiments aiming to unravel the intimate molecular mechanisms involved. To our knowledge, this ‘micro-ecological’ perspective of the immune interactions has not been proposed so far for any other blood-feeding parasite and associated pathogens. In systems, such as the honeybee–*Varroa* mites interaction where the parasite removes a substantial amount of blood from the host, this model could probably play a significant role. Thus, these results lay the groundwork for future research into the role of these predator–prey dynamics in other systems, and studies of the underlying molecular and physiological mechanisms. Furthermore, this study provides key insights into the crucial role played by *Varroa* mite in the re-emergence of DWV, an endemic pathogen of honeybees that plays a key role in the current widespread crisis of the beekeeping industry.

## 4. Material and methods

### (a) Viral infection in mite-infested bees and mites, and effects on bees' transcriptome

In order to study DWV infection in bees and the infesting mites, we artificially infested honeybees from our experimental apiary [35] (see the electronic supplementary material, Material and methods for more details) with one or no mites as previously described [36] (see the electronic supplementary material, Material and methods for more details, including sample sizes). Sequencing of DWV, quantitative DWV analysis, analysis of DWV mutant cloud, DWV negative strand quantitative analysis and the transcriptomic study of bees were carried out using standard methods. Briefly, quantitative DWV analysis was carried out by means of qRT-PCR, using sequence-specific primers, whereas all other analyses were performed by NGS techniques, described in detail in the electronic supplementary material, Material and methods. As a rule, transcriptomic analyses were carried out on five samples per experimental group; full-length genome sequencing of the virus was done on the genetic material obtained from two highly infected bees, whereas the study of the viral mutant cloud is based on 10 virus infected bees.

DWV concentration in the honeybee haemolymph was quantified as described above on a sample obtained as described in electronic supplementary material, Material and methods.

### (b) Role of haemocytes in the antiviral response of honeybees

To assess if haemocytes are involved in antiviral response in honeybees, we saturated phagocytosis by injecting a suspension of chromatography beads into white-eye pupae, naturally infected by DWV, and measured the viral load 48 h later.

White-eye honeybee pupae were manually extracted from a sealed brood comb, taken from a colony at the end of autumn when, according to previous studies, virus prevalence reaches 100% in all colonies [9]. CM Sepharose fast flow chromatography beads (Pharmacia), suspended in 2 µl of phosphate buffered saline, were injected into the haemocoel of honeybees using a Hamilton syringe equipped with a sterile 30 gauge needle. Then bees were maintained on sterile filter paper in small Petri dishes in an incubator (34°C, 75% relative humidity (R.H.), dark).

After 48 h, 10 bees per experimental group were sampled for DWV quantification by qRT-PCR; a few other bees were sampled for microscopic analysis. The experiment was carried out once using 40 bees per experimental group.

### (c) Study of the effects of an increasing haemolymph subtraction on viral replication

This experiment was designed to assess the effect of the removal of an increasing haemolymph volume, in the absence of feeding mites, on the dynamics of DWV titre in naturally infected honeybees. Last instar bee larvae were collected from a brood comb as described above and maintained in an incubator (34°C, 75% R.H., dark) until the white eyes stage, which occurred about 4 days after the collection from brood cells sealed in the preceding 15 h. Then, four experimental groups, made of about 30 pupae each, were established. One group (control) was left untreated, whereas all the other bees had the right antenna cut, at the level of the scapum, using fine scissors; pupae bleeding after cutting were discarded. Bees of one group (wound) had the wound sealed with a cream containing Sulfathiazole (2%) and Neomycin sulfate (0.5%) to prevent secondary infections. Bees of the remaining two groups (wound −1 µl’ and ‘wound −2 µl) had the wound sealed as above, after removing either 1 or 2 µl of haemolymph, with a microcapillary tube precisely graduated with 1 or 2 µl of ethanol, dispensed through a micropipette. By subtracting increasing amounts of blood, we tried to assess the effect of pure haemolymph subtraction, while maintaining constant the impact of wounding and the resulting immune reaction.

The choice of the volume of haemolymph to be subtracted was dictated by available data showing that *V. destructor* can consume as much as 0.7 µl of bee haemolymph every 24 h [25], which, according to our data, can contain up to 10^3^–10^8^ DWV particles µl^−1^ according to the infection level.

After treatment, bees were kept in a Petri dish, lined with sterile filter paper and maintained under dark, at 34°C, 75% R.H., until eclosion. After 4 or 8 days, 10 bees from each experimental group were sampled to assess the viral titre as described below. To account for the variability across colonies and genotypes, the experiment was repeated four times: on two colonies in Udine (Northern Italy) and two colonies in Napoli (Southern Italy).

Ten bees from each experimental group, from the second replicate of the experiment, carried out in one location, were also used to assess the expression level of *Dorsal 1A*, a gene in the NF-kB family, indicating an active immune suppression by the DWV [9]. Sample processing was as explained below whereas dorsal analysis was carried out as described in ref. [9].

### (d) Statistical analysis

The statistical analysis of data was carried out using standard methods [37,38] described in detail in the supplementary material, Material and methods.

### (e) Simulations

In [9], we presented a series of models capturing the coupled within-host dynamics of viral copy number (*V*) and a shared immune currency (*I*). The most parsimonious model analysed further in the current paper included an immunosuppressive effect of high viral load, as described by the following ordinary differential equations

4.1 $$\begin{array}{rl} \frac{dV}{dt}=\; & (1-I)V\\ \mathrm{and}\;\frac{dI}{dt}=\; & x-yI+z(1-V)V. \end{array}\}$$

These equations describe the within-host growth of a pathogen population *V* and its controlling immunological counterpart *I*. In this dimensionless form [9], the units of time are rescaled to the maximal growth rate of the virus, the units of viral density to the density that halts immune proliferation and the units of immune density to the density that halts viral proliferation. The dynamics of *V* is shaped by the maximal rate of pathogen replication (scaled to 1), which is counteracted by immunological control. The dynamics of *I* are shaped by an intrinsic production rate *x*, a rate of decay *y* and an activation/suppression parameter *z.* This model implementation ensures that the sign of the impact of the virus on immune dynamic (immunostimulatory or immunosuppressive) will depend on viral titre, *V*. Specifically, we assume that at low densities, the pathogen is a net activator of immunological activity, whereas at high densities (whenever *V* > 1), the pathogen becomes immunosuppressive.

To study the impact of an episodic removal of haemolymph on viral level, we conducted phase portrait and time-series analyses of equations (4.1) under differing scenarios of haemolymph removal and concurrent initial reductions in both viral and immunological titres *V* and *I.* In this case, we applied a 20% drop in haemolymph volume which is consistent with available data on the reduction observed in mite-parasitized bees [25,26].

## Supplementary Material

Annoscia Supplementary information

## Supplementary Material

Supplementary Data 1

## Supplementary Material

Supplementary Data 2

## Supplementary Material

Supplementary Data 3

## Supplementary Material

Supplementary Data 4

## Data accessibility

This article has no additional data.
