# Supplementary material for: Haemolymph removal by Varroa mite destabilizes the dynamical interaction between immune effectors and virus in bees, as predicted by Volterra's model
Source: Proc Biol Sci. 2019 Apr 17;286(1901):20190331. doi: 10.1098/rspb.2019.0331 (PMC6501932; doi:10.1098/rspb.2019.0331)
Supplement: Annoscia Supplementary information [file rspb20190331supp1.docx]

Haemolymph removal by *Varroa* mite destabilizes the dynamical interaction between immune effectors and virus in bees, as predicted by Volterra’s model

Desiderato Annoscia^1^, Sam P. Brown^2^, Gennaro Di Prisco^3,4^, Emanuele De Paoli^1^, Simone Del Fabbro^1^, Davide Frizzera^1^, Virginia Zanni^1^, David A. Galbraith^5^, Emilio Caprio^3^, Christina M. Grozinger^5^, Francesco Pennacchio^3^, Francesco Nazzi^1^

^1^Dipartimento di Scienze AgroAlimentari, Ambientali e Animali, Università degli Studi di Udine, Udine, Italy.

^2^School of Biological Sciences, Georgia Institute of Technology, Atlanta, GA, USA.

^3^Dipartimento di Agraria “Filippo Silvestri”, Università degli Studi di Napoli “Federico II”, Portici (Napoli), Italy.

^4^CREA, Council for Agricultural Research and Economics, Research Center for Agriculture and Environment, Bologna, Italy.

^5^Department of Entomology, Center for Pollinator Research, Huck Institutes of the Life Sciences, Pennsylvania State University, University Park, PA, USA.

Corresponding author: Francesco Nazzi, email: [francesco.nazzi@uniud.it](mailto:francesco.nazzi@uniud.it)

Doi: 10.1098/rspb.2019-0331

**Supplementary figures**

**Figure S1. Phylogenetic relationship of the DWV infecting the bees used in this study (samples P11 and P31).** The dendrogram is based on the complete genomes (nt sequences) of available sequences of DWV strains and related viruses.

**Figure S2. DWV prevalence in bees infested or not by a DWV infected mite.** Error bars represent the estimated confidence limits of the proportion.

**Figure S3. Number of samples with a given infection levels in bees infested with one mite in late summer.**

**Figure S4. Viral load in individual bees artificially infested at the L5 stage with one, three or no mites and sampled after 1, 6 and 12 days (eclosion).** A further sample, infested by 3 mites and collected 1 day after eclosion, with infection level=1.22E+03, does not appear on this graph. The horizontal bars represent the median infection level.

**Figure S5. Infection level in infesting mites and infested bees.** Note that zero values were transformed to 1.

**Figure S6. Diversity of the mutant cloud, as estimated with the Shannon index, and viral infection in bees.**


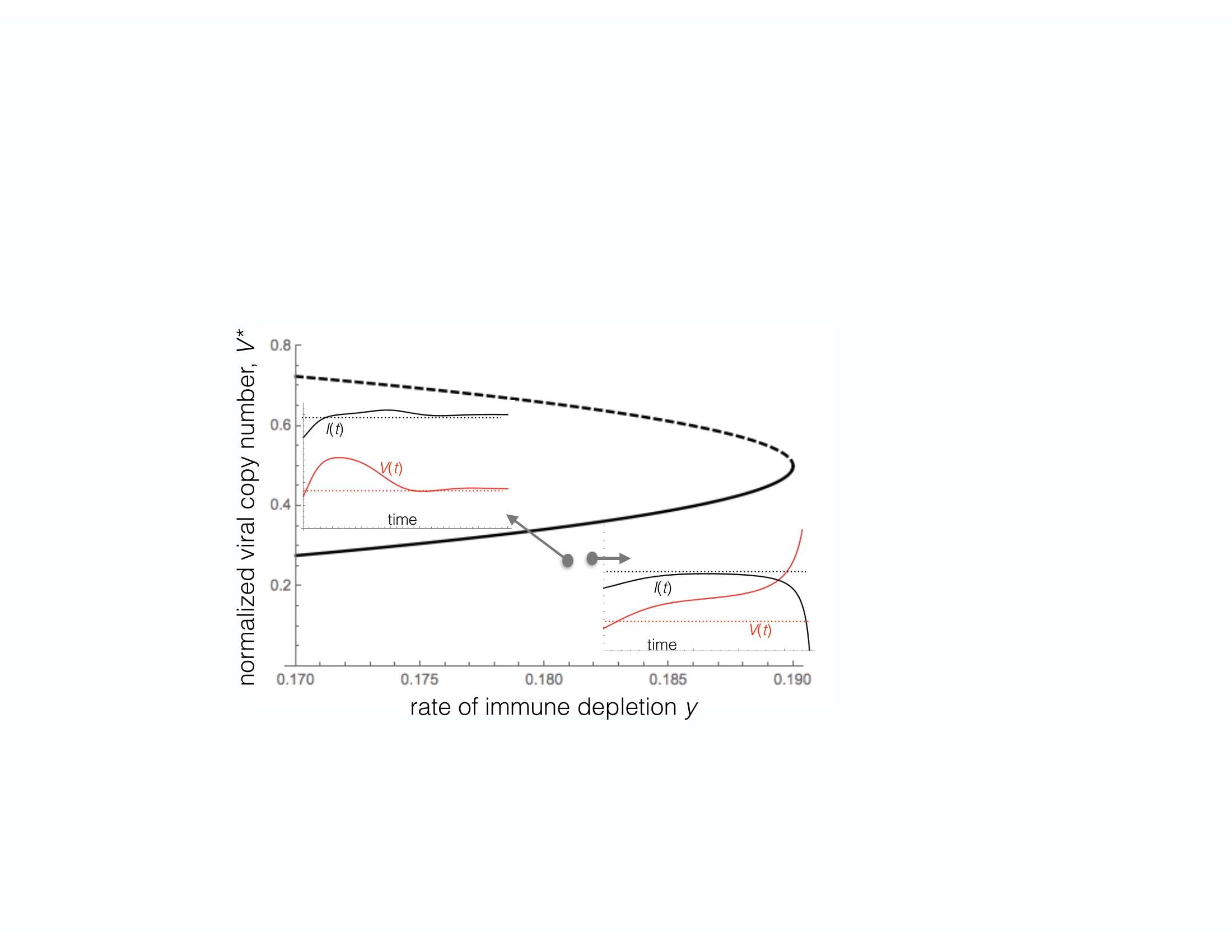


**Figure S7. Haemolymph loss destabilises viral dynamics.** Stable (solid line) and unstable (dashed line) viral titre equilibria *V** are plotted as a function of increasing immune depletion *y.* The lower stable equilibrium is {*V* =* $\frac{1}{2}-\frac{\sqrt{z+4(x-y)}}{2\sqrt{z}}$, *I* =* 1}. For model derivation and analysis, see ref. 9. Above the dashed line (and for high *y*, any point to right of intersection with solid line), the virus cannot be efficiently regulated and a viral explosion ensues. To explore the impact of perturbations around the lower stable equilibria, we conducted numerical experiments varying the initial conditions as *V*(0) and *I*(0), as proportional deviations from the stable equilibrium {*V* =* $\frac{1}{2}-\frac{\sqrt{z+4(x-y)}}{2\sqrt{z}}$, *I* =* 1}. The two dots represent a 20% drop below {*V*, I**}, and the two insets illustrate the corresponding time series of *V*(*t*) and *I*(*t*) (red and black), relative to the stable equilibrium values (dotted lines). Parameters are the same as for ref. 9: *x* = 0.09, and *z* = 0.4.

**Figure S8. Haemocytes contribute to antiviral defence in honey bees.** (A) Chromatographic beads injected into bee pupae were encapsulated by haemocytes. (B) Saturating phagocytosis in PBS+beads treated bees caused a significant increase in viral load as compared to bees treated with PBS only.

**Figure S9. Differential immune response in bees infested by no, one or three mites.** (A) For each contrast between treatments, the number of differentially expresses genes belonging to the canonical immune pathways is reported. (B) Clustering of individual bees infested by no mites, one mite, or three mites according to the expression level of immune genes.

**Figure S10. Average expression level of Dorsal 1A in bees exposed to different treatments.** The error bars represent the standard deviation.

**Fig. S11. DWV genome copies in bees 8 days after the removal of 1 or 2 µL of haemolymph as compared to wounded bees with no haemolymph subtraction and control bees receiving no treatment.** Different letters mark experimental groups significantly differing from each other (*P*<0.001, capital letters; *P*<0.05, lowercase letters); consistently with the statistical analysis used here, the horizontal solid lines represent the average viral load.

**Figure S12. Possible routes of infection of DWV.** The bees are represented by squares of different shades of blue according to the developmental stage (dashed arrows highlight the development of the bee), the virus is a yellow pentagon and the mite an orange hexagon. Both trans-ovarial (from queen to egg, 1) and trans-stadial (from egg to larva, 2, and from larva to adult, 3) as well as horizontal transmission between nurse bees and larvae (4) and from adult to adult (5) are possible among bees (blue arrows highlight bee mediated infection routes). The *Varroa* mite can transfer the virus from pupa to pupa (6), pupa to adult (7) and viceversa (8) and adult to adult (9). Note the possible infection routes of the bee larva (2, 4), that are possible independently of the mite’s presence. The contribution of the mite as a virus vector is highlighted by transmission routes 6, 7, 8, 9 (red arrows).

**Figure S13. Current hypotheses about the role of the mite in facilitating viral infections**. The bees are represented by squares, the virus is a yellow pentagon and the mite an orange hexagon; violet triangles (empty after suppression) represent the immune effectors. (A) The virus replicates within the mite and subsequently infects the pupa [8]. (B) The mite favours the proliferation of a virulent strain of the virus (in the case represented here as an example, by injecting the pathogen into the haemolymph where replication is easier) [11, 12]. (C) The mite suppresses the bee’s immune response [10]. (D) The mite engages the same factors needed to sustain the antiviral response, releasing the pathogen from immune control [9]. (E) The mite feeds upon the virus-contaminated haemolymph, subtracting both DWV and immune effectors, altering the dynamics of the systems, resulting in increased virus abundance (this article).

**Supplementary Materials and Methods**

**Bees and mites used in this study.** The biological material (honey bee worker larvae and *Varroa destructor* adult females) was obtained from an experimental apiary located in Udine (Northeastern Italy). Previous studies indicated that local colonies are hybrids between *Apis mellifera ligustica* Spinola and *Apis mellifera carnica* Pollman [35].

**Artificial infestation of bees with *V. destructor* mites.** The bee larvae and the mites were collected from brood cells capped 0-15 h previously. Larvae obtained as above were transferred into gelatine capsules (Agar Scientific Ltd., 6.5 mm diameter), artificially infested with one mite or left uninfested, and maintained in an incubator (34 °C, 75% R.H., dark) until the adult emergence [36] (Fig. 1A). Newly emerged adult bees and the infesting mites were stored at –80 °C for subsequent molecular analyses. Sixty bees per experimental group were prepared with this method, which, after removing dead bees and mites, resulted in 40 uninfested honey bees (32 DWV positive) and 32 infested honey bees (30 DWV positive) together with 32 infesting mites (27 DWV positive), that were used in the analysis (Fig. 1B). To confirm the results on the distribution of viral infection levels in mite infested bees, another 58 infested bees, prepared as above, were analysed subsequently.

In order to test the relative importance of immunity on the increased viral titer normally observed in multiple infested bees, we artificially infested with no, one or three mites five last instar honey bee larvae obtained as described above and compared the expression of immune genes in those bees, as described below. Furthermore, to assess if increased viral replication observed in multiple infested bees may be related to the reduced quality of the host and, in particular, to the loss of nutrients, we also compared the expression of genes involved in metabolism and nutrient utilization.

**Quantification of DWV concentration in the honey bee haemolymph.** Haemolymph was collected from late 5th instar honey bee larvae by puncturing with an entomological needle the dorsal vessel and collecting the bleeding fluid with a capillary tube. Haemolymph was transferred to an Eppendorf tube containing the same quantity of anti-coagulant buffer [39], mixed and centrifuged at 1,000 g for 10 min at 4 °C. Then, the supernatant containing the plasma was discarded and DWV quantified as described below.

**Sequencing of DWV.** The de novo assembly of DWV was performed using CLC Genomics Workbench 9.0. Briefly, simple contig sequences were first created using the information from the read sequences using an assembly algorithm based on de Bruijn graphs -a compact representation based on short words (k-mers) that is ideal for high coverage, very short read (25-50 bp) data sets- then all reads were mapped using the simple contig sequence as a reference.

A phylogenetic tree was constructed from two sequences obtained in this study (samples P11 and P31, Figs. 1B-1E) and 12 available complete DWV genomes including:

gi|71480055|ref|NC_004830.2| Deformed wing virus, complete genome,

gi|31540603|gb|AY292384.1| Deformed wing virus isolate PA, complete genome,

gi|390190253|gb|JQ413340.1| Deformed wing virus isolate Chilensis A1, complete genome,

gi|592965310|gb|KJ437447.1| Deformed wing virus isolate Varroa-infested-colony-DJE202, complete genome,

gi|40714033|dbj|AB070959.1| Kakugo virus genomic RNA, complete genome,

gi|47177088|ref|NC_005876.1| Kakugo virus, complete genome,

gi|430007848|gb|JX878304.1| Deformed wing virus strain Korea-1, complete genome,

gi|430007850|gb|JX878305.1| Deformed wing virus strain Korea-2, complete genome,

gi|56121875|ref|NC_006494.1| Varroa destructor virus-1, complete genome,

gi|55925812|gb|AY251269.2| Varroa destructor virus 1, complete genome,

gi|301070167|gb|HM067437.1| Deformed wing virus isolate VDV-1-DWV-No-5, complete genome,

gi|301070169|gb|HM067438.1| Deformed wing virus isolate VDV-1-DWV-No-9, complete genome.

Multiple sequence alignment was performed using the ClustalW algorithm and the phylogenetic tree was constructed with “MEGA7: Molecular Evolutionary Genetics Analysis version 7.0 for bigger datasets” [40] using the neighbor-joining method; bootstrap values are based on 1000 replicates.

**Quantitative DWV analysis.** Total RNA was isolated from individual honey bees using TRIzol reagent (Invitrogen), according to the manufacturer’s instructions. The concentration and purity of total RNA were determined by spectrophotometry (Varioskan Flash Spectral Scanning Multimode Reader; Thermo Fisher Scientific).

The quantification of DWV genome copies was performed by SYBR Green qRT-PCR. Titers of DWV were determined by relating the Ct values of unknown samples to an established standard curve. The standard curve was established by plotting the logarithm of seven 10-fold dilutions of a starting solution containing 21.9 ng of plasmid DNA pCR II-TOPO (TOPO-TA cloning) with a DWV insert (from 21.9 ng to 21.9 fg), against the corresponding Ct value, as the average of three repetitions.

The PCR efficiency (E=107.5%) was calculated based on the slope and coefficient of correlation (R^2^) of the standard curve, according to the following formula: E=10(−1/slope)−1 (slope=−3.155, y-intercept=41.84, R^2^=0.999). Amplifications were performed using the StepOne Real-Time PCR System (Life Technologies) with the following thermal cycling profiles: one cycle at 48 °C for 15’ for reverse transcription, one cycle at 95 °C for 10’; 40 cycles at 95 °C for 15’’, 60 °C for 1’; one cycle at 68 °C for 7’, using the Power SYBR Green RNA-to-Ct 1-Step Kit (Thermo Fisher Scientific). Primer pair (DWV-Forward 5’-GCGCTTAGTGGAGGAAATGAA-3’; DWV-Reverse 5’-GCACCTACGCGATGTAAATCTG-3’) was designed using PrimerExpress 3.0 software (Life Technologies) following the standard procedure. Negative (H_2_O) and positive controls (previously identified positive samples) were included in each qRT-PCR run. According to the manufacturer, the used equipment should allow the detection of the virus provided that at least 50 genome copies are present in the sample.

**Analysis of DWV mutant cloud.** For DWV RNA-dependent RNA polymerase (DWV\RdRp) analysis, specific primer pair were chosen for the amplification of a genome portion of 454 bp (DWV\RdRp-Forward 5’-TAGTGCTGGTTTTCCTTTGTC-3’; DWV\RdRp-Reverse 5’-CCCAGGACCAAAATTCTTAT-3’). The PCR reactions were conducted using the Invitrogen SuperScript III One-Step System (Platinum) following the manufacturer’s procedure. The thermal profile was: 50 °C for 30’; 94 °C for 2’; 40 cycles at 94 °C for 15’’, 60 °C for 30’’, 68 °C for 1’; 68 °C for 5’. Amplified products were run on a 1% agarose gel containing 0.5 µg/mL ethidium bromide and then visualized by UV transillumination. The specificity of the RT-PCR assay was confirmed by sequencing analysis. RT-PCR bands were excised from the agarose gel and purified using the Pure Link Quick Gel Extraction Kit (Invitrogen, Carlsbad, CA). The sequence data of the fragment were analysed using the BLAST server at the National Center for Biotechnology Information, NIH. For the RNAseq analysis, the same primer pairs described above were designed with a specific overhang (DWV\RdRp-Forward 5’-TCGTCGGCAGCGTCAGATGTGTATAAGAGACAG-3’; DWV\RdRp-Reverse 5’-GTCTCGTGGGCTCGGAGATGTGTATAAGAGACAG-3’). All primer pairs were designed by using Primer Express Software (Applied Biosystems).

Raw Illumina paired-end reads in forward and reverse orientation were merged using the PEAR software v0.9.8 [41], adaptor trimmed using cutadapt v1.9 [42] and quality filtered using the FASTX Toolkit’s fastq_quality_filter program [43] requiring minimum quality of 20 in 95% bases or more.

For the study of DWV variants we used a software designed for quasi-species reconstruction from next-generation sequencing data [44]. Sequenced fragments were aligned against a reference genome and the reference genome was partitioned into a set of sliding windows, then a reconstruction algorithm based on combinations of multinomial distributions was applied. The selected reference genome was the one most closely related to the DWV samples sequenced in this study (i.e. NC_004830.2; Fig. S1). The analysis output is a collection of sequences with prevalences, which was used for a diversity analysis based on the Shannon index.

**DWV Negative strand quantitative analysis.** In order to quantify the DWV negative strand in infesting mites, a strand-specific Biotin/Streptavidin method was used. SYBR-Green real-time quantitative PCR (qPCR) as described above was preceded by a specific retrotranscription incorporated with biotinylated-primers (Biotin-DWV-Forward 5’-TCG ACA ATT TTC GGA CAT CA-3’; Biotin-DWV-Reverse 5’-ATC AGC GCT TAG TGG AGG AA-3’) and magnetic bead purification by using the Dyna beads KilobaseBINDER Kit following the manufacturer’s instructions (Applied Biosystems).

**Transcriptomic study of bees.** For this purpose, five uninfested and 10 newly emerged infested bees (5 with a high DWV infection level and 5 with a low infection level) were used (Fig. 1B). Also, five newly emerged bees infested with no, one or three mites were processed as described below.

Samples were transferred into liquid nitrogen and the total RNA isolated using Tri-reagent (MRC Inc., USA) (Ambion Inc.). RNA sequencing libraries were generated starting from 1-2 micrograms of high quality RNA (RNA Integrity Number >7, Agilent Technologies Bioanalyzer, Agilent Technologies, USA) by means of TruSeq Standard mRNA seq kit Illumina, following the standard protocol indicated by the producer (Illumina, Inc., CA, USA). This protocol produced 25-30 million reads per sample that were 36 bases in length.

The sequencing reads were trimmed using ERNE [45] in order to remove low quality reads and the adapters removed with Cutadapt [42]. The remaining reads were aligned to the most recent honey bee genome build (Amel 4.5 [46]) with TopHAt2 [47] using default parameters and annotated with the newest official gene set (OGS 3.2). Reads were counted with Htseq count [48] using exons as accepted regions and cumulating the counts at whole gene level.

The obtained data were uploaded into DEseq2 [49] to be elaborated by means of VST (Variance Stabilizing Transformation) algorithm and used for the cluster analysis.

To gain insight into the relative contribution of the *Varroa* mite and DWV in the alteration of the expression of genes belonging to the canonical immune pathways, we studied the transcriptome of bees exposed to a different combination of stress factors (Supplementary Data 2). In particular, to assess the influence of the mite, we compared the expression level of 191 immune genes, listed in ref. 12, in five uninfested bees bearing a low viral infection (average DWV infection=2.04E+03) and five mite infested bees bearing a similar, low viral infection level (average DWV infection=1.95E+03) (Fig. 1B); instead, to assess the influence of the combination *Varroa*-DWV we compared five uninfested bees bearing a low viral infection with five mite infested bees bearing a high viral infection level (average DWV infection=1.41E+09) (Fig. 1B). FPKM were used as gene expression data. The list of honey bee immune genes proposed by Ryabov et al. [12] was used (note that 14 and 151 genes classified in ref. 12 as “immune related” and “immune system process” respectively, were not included in this analysis that concentrated on canonical immune pathways).

Due to the uncertainty about the normality of data distribution, the statistical testing of the significance of differences was carried out using the Mann-Whitney U test. Since the purpose of this study was not to gather data about single genes but rather to gain an indication about the involvement of different immune pathways, and keeping into account the limited probability of type I errors, no correction for multiple comparisons was applied but rather differentially expressed genes were considered as such when probability was below 0.01. In fact, since the list of differentially expressed genes was compiled from a total of 162 genes, the number of possible false positives was less than two and therefore, the probability that this could affect the results of the subsequent analysis we carried out (i.e. comparison of different canonical immune pathways) was negligible. To test this assumption, we assessed the possible impact of type I errors on our analysis by means of resampling. To this end, we subtracted one differentially expressed gene (i.e. a gene that could be regarded as a false positive) from a randomly selected pathway among those including the DEGs and recalculated the significance of the enrichment. After replicating this procedure 1000 times, we found that the presence of 1 false positive could result in a different conclusion of our enrichment analysis in less than 0.1 % of cases, both when testing the mite effect and the mite plus virus effect.

To verify the possible enrichment of each immune pathway, the proportion of differentially expressed genes belonging to that pathway out of the total number of DEGs in that contrast was compared with the proportion of genes belonging to that pathway out of the total number of genes considered here; a Chi square test was used for the statistical analysis and the significance was set again at 0.01. A Benjamini-Hochberg correction for multiple comparisons was used, setting the false discovery rate at 0.05.

In order to test the relative importance of immunity and nutrient loss on the increased viral titer normally observed in multiple infested bees, we artificially infested with none, one or three mites five last instar honey bee larvae obtained as described above and compared the expression of immune genes and genes involved in nutrient utilization in those bees, as described above (Supplementary Data 3 and Supplementary Data 4). FPKM were used as gene expression data. The list of honey bee immune genes proposed by Ryabov et al. [12] was used; the list of genes involved in nutrient utilization was obtained from Foret et al. [50]. The proportion of reads mapping onto the DWV genome provided an estimation of the viral infection level in bees.

To remove possible outlier samples from the analysis, the mean expression value across all samples for each gene was calculated as well as the standard deviation (note that here samples were considered as belonging to a single big group, in order not to introduce any bias in our preliminary filtering). Then expression values higher or lower than the mean expression value ±2 standard deviations were noted. Lastly the proportions of outlier genes in each sample were considered and samples having more than 25% of outlier genes were excluded (this procedure lead to the exclusion of one single sample out of 15, which appeared to have a number of outlier genes bigger by three fold than any other sample).

The statistical testing of the significance of difference among groups was carried out using the Mann-Whitney U test; a Benjamini-Hochberg correction for multiple comparisons was used, setting the false discovery rate at 0.1.

The cluster analysis reported in figure S9B was carried out using “Gene Cluster 3.0” [51] with the following options: centering genes around mean, similarity metrics: correlation (uncentered), clustering method: average linkage. The tree was displayed using “Treeview” [52].

**Statistical analysis of experimental data.** The proportion of infected bees (Fig. S2) was compared by means of a Chi Square test; the confidence intervals reported in the figure were calculated with the formula: 1.96√(prop(1-prop)/n). The skewness of the distribution of viral loads across samples was calculated after excluding samples where the virus had not been detected.

Since the normality of the distribution of data about viral infection in bees, as assessed by real time RT-PCR, is not warranted, non-parametric methods were preferred to standard parametric analysis. In particular, the correlation between viral load in mites and bees infested by those mites (Fig. S5) was tested by means of Spearman rank. Comparison between infection levels in bees belonging to different experimental groups was carried out by means of a Mann-Whitney U test (Figs. 1B, 1C, 1D) or Kruskal-Wallis (Fig. 2A). The results reported in figure 3 and figure S11 were first log transformed and then analysed by means of Linear mixed effect using R [37], lme4 [38], with treatment as a fixed effect and location and replication as random effects; P value was obtained by likelihood ratio tests of the full model with the effect in question against the model without the effect in question. A Tukey test was used for the post-hoc comparison of the differences among experimental groups. In this experiment, aiming at studying the effect of the treatment on viral replication, DWV negative samples were not considered.
